# The Role of Place in Shaping Contraceptive Use among Women in Africa

**DOI:** 10.1371/journal.pone.0040670

**Published:** 2012-07-18

**Authors:** K. Miriam Elfstrom, Rob Stephenson

**Affiliations:** Hubert Department of Global Health, Rollins School of Public Health, Emory University, Atlanta, Georgia, United States of America; Tehran University of Medical Sciences, Iran (Republic of Islamic)

## Abstract

**Background:**

Contraceptive prevalence is low in the African region despite considerable family planning programmatic efforts. This study is the first to examine how community factors shape contraceptive use for married women in an entire region, comparing results across 21 African countries with a DHS in the last 5 years. The analysis builds on previous studies through an examination of the individual, household and community level factors that shape contraceptive use.

**Methods:**

The data used in this analysis were from nationally representative Demographic and Health Surveys completed between 2005 and 2009. A separate multi-level logistic model was fitted for the outcome of current modern contraceptive use in each country.

**Results:**

After controlling for individual and household level factors, community level factors of demographics and fertility norms, gender norms and inequalities, and health knowledge remain significantly associated with contraceptive use, although the magnitude and direction of these community effects varied significantly across countries.

**Conclusions:**

The results highlight the importance of harnessing community level factors in planning interventions for increasing access to and utilization of modern contraceptive methods.

## Introduction

Significant variations in contraceptive prevalence exist in the African region. In Eastern Africa, among women who are married or co-habitating, contraceptive prevalence ranges from 10.8% in Rwanda to 58.0% in Zimbabwe whereas in Western Africa, the range is much smaller (5.4% in Guinea to 16.5% in Ghana) (2005 Rwanda DHS, 2005–06 Zimbabwe DHS, 2005 Guinea DHS, 2008 Ghana DHS). This variation in prevalence also reflects significant differences in method profiles between countries [1]. While contraceptive prevalence has increased steadily by 1–2% in some countries, other countries continue to lag behind. Contraceptive prevalence in Benin, Guinea, Mali, Niger, Nigeria, Senegal, and Sierra Leone has increased by less than 0.5% per year since 1997 [1]. Contraceptive uptake has been positively associated with a range of both health and non-health related outcomes. However, despite the impact that family planning can have on preventing HIV transmission, helping women achieve their desired fertility, and increasing women’s empowerment, contraceptive prevalence remains uneven [2], [3], [4]. Programmatic efforts to increase uptake have not successfully reached all segments of the population, leaving women in rural areas and poorer women behind [5].

Previous research has explored modern contraceptive use in resource-poor settings and some studies have examined the impact of contextual factors on contraceptive use. However, studies have been narrow in their geographic reach as they have focused on a limited number of countries or communities [6], [7], [8], [9]. Studies have concentrated on fewer community level variables and have taken a particular interest in examining the impact of socio-economic status, supply environment, and quality of care when examining how where a woman lives shapes her health outcomes [10], [11]. This paper investigates associations between community level factors and current modern contraceptive use in all 21 African countries with completed Demographic and Health Surveys (DHS) from 2005–2009. Community level variables included in this analysis move beyond factors explored in the past – such as factors related to the health infrastructure of a community – to describe how community context influences individual health outcomes. This is the first study of its kind to examine community level influences on contraceptive use for an entire region and capture a broader range of contextual factors. Through examining the influence of community demographics and fertility norms, economic prosperity, gender norms and inequalities, health knowledge, and media exposure this study seeks to capture the influence of social space in shaping contraceptive use [12]. Identifying community level factors associated with contraceptive use is critical to informing family planning programmatic efforts and understanding how community environments shape contraceptive uptake.

There has been a growing interest in examining how community level factors shape health outcomes [13], [14], [15]. The emergence of multi-level modeling as a technique for capturing the effect of community level factors allows for analyzing hierarchically clustered data and estimating variation between communities [13], [14], [15], [16], [17]. This is important because individuals in the same community are more likely to be similar than individuals in different communities [16], [17]. If this clustering is ignored, the standard errors are underestimated. Multilevel modeling corrects the estimated standard errors resulting from this clustering [16]. By including contextual influences, risk factors for adverse health outcomes associated with specific community characteristics can be determined and public health interventions developed that are adapted to community level needs [17].

The connection between community level factors and reproductive health outcomes has been demonstrated in previous studies, highlighting the importance of place in shaping individual’s health outcomes. In the past, research has emphasized the presence and quality of the health infrastructure in a community and the socio-economic status of the community as influential community level factors [18], [19], [20], [21]. Women in communities with stronger health service presence were more likely to seek reproductive health care services [21]. Additionally, women’s decisions are influenced by the accessibility of health services in the community and by the general socio-economic status of the community as stronger health infrastructure and higher socio-economic status decrease logistical barriers to seeking services [9], [10], [22], [23]. Stronger health system level presence in a community translates into more opportunities to build awareness of family planning and confidence in services provided [24].

With regard to the role that community level fertility norms play in shaping individual contraceptive uptake, several studies have demonstrated that women are influenced by perceived community fertility expectations and commonly held beliefs regarding side-effects of modern contraceptives [8], [23], [25], [26]. Specifically, women in communities with a higher mean number of births per woman compared to women in communities with a lower mean number of births per woman have been shown to be less likely to use a contraceptive method [8]. The mean age at first sexual intercourse is also associated with contraceptive use and, in particular, with type of method used [27]. Women’s reproductive health decisions – including the choice to use contraception – are shaped by the norms and beliefs of the community in which they live and also by the general level of autonomy experienced by women in the community [22], [26], [28].

Educational attainment is associated with modern contraceptive use at the community level where both the mean number of years of schooling and the proportion of women with at least a primary education have been shown to be positively associated with family planning uptake [23], [27]. With regard to exposure to external sources of information and health knowledge the community level, previous studies examining community level influences on modern contraceptive use have shown a positive association between exposure to media messages regarding family planning and contraceptive use [6], [8]. Taken together, greater educational attainment and media saturation at the community level may relate to increased levels of health knowledge and household wealth facilitating greater autonomy in seeking health care services. Women’s autonomy in seeking health services may be further impacted by experiencing domestic violence. Studies have shown an association between experience of intimate partner violence and contraceptive use that is somewhat complex [22], [29], [30]. Diop-Sidibe et al. found a negative association between contraceptive use (women are less likely to use a contraceptive method if they had experienced domestic violence) whereas Stephenson et al. found that women in communities where a higher proportion of women reported experiencing physical violence from their partner are more likely to use a contraceptive method. As Diop-Sidibe et al. suggest, women may feel that their health seeking behavior is more controlled by their partner; however, they may also feel that their health status is poorer and therefore seek additional reproductive health services.

In general, studies investigating community level influences on reproductive health care seeking behavior have focused primarily on the availability of services in the community and the socio-economic status of the community [10], [15], [19], [20], [21], [31], [32]. Studies have tended to focus on a limited number of community influences and many have focused on one country of analysis [6], [8], [9]. Very few studies have examined a broader range of factors, especially the influence of fertility norms, gender norms and inequalities, and health knowledge. This study includes an expanded range of community level factors and offers a cross-national comparison of 21 different countries. By comparing distinct country settings in the African region, the results of this analysis will contribute to a deeper understanding of the community level factors associated with contraceptive use.

## Methods

The data used in this analysis were from nationally representative Demographic and Health Surveys (DHS) from 21 African countries. All countries that had a DHS completed between 2005–2009 were included to capture the most current data on contraceptive use: Benin (2006), Congo (2005), Democratic Republic of Congo (2007), Egypt (2008), Ethiopia (2005), Ghana (2008), Guinea (2005), Kenya (2008–2009), Liberia (2007), Madagascar (2008–2009), Mali (2006), Namibia (2006–2007), Niger (2006), Nigeria (2008), Rwanda (2005), Senegal (2005), Sierra Leone (2008) Swaziland (2006–2007), Uganda (2006), Zambia (2007), Zimbabwe (2005–2006). The DHS were carried out by ORC Macro in partnership with local governments and institutions. The sampling systems used in each country were similar and were based on a two-stage sampling design. In the first stage, Primary Sample Units (PSUs) were selected using the most recent census in each country as the sample frame. Approximately 20 to 30 households were then selected from a listing of households in each PSU. All ever-married women of reproductive age (15–49) were eligible to be included. For this analysis, the samples were limited to currently married or co-habitating women. The resulting sample sizes are shown in Table 1, grouped by region. Overall response rates for the women’s survey were high and ranged from 90.2% (Zimbabwe) to 99.7% (Egypt). Data on fertility, family planning, and health knowledge as well as demographic and socioeconomic information were collected. The study was approved by the Emory University Institutional Review Board. The study uses secondary data collected through the Demographic and Health Survey program. The data is publicly available for download from www.measuredhs.com. Written consent was taken in the collection of the original data. Further information about the details of the survey content and methodology are available at http://www.measuredhs.com/.

**Table 1:** Sample sizes and proportion of current modern contraceptive use among women (15–49) in 21 study countries.

Region	Country (Year)	Total SampleSize (N)	Currently Married orCo-Habitating Women(unadjusted) (n)	Current modern contraceptive use (unadjusted) (%)
Eastern Africa				
	Ethiopia (2005)	14,07	8,644	16.0
	Kenya (2008–09)	8,444	5,041	36.0
	Madagascar (2008–09)	17,375	11,903	28.4
	Rwanda (2005)	11,321	5,458	10.8
	Uganda (2006)	8,531	5,362	17.0
	Zambia (2007)	7,146	4,316	33.1
	Zimbabwe (2005–06)	8,907	5,118	58.0
Middle Africa				
	Congo (2005)	7,051	3,993	13.2
	Democratic Republic of Congo (2007)	9,995	6,586	6.4
Northern Africa				
	Egypt (2008)	16,527	15,406	55.3
Southern Africa				
	Namibia (2006–07)	9,804	3,578	51.6
	Swaziland (2006–07)	4,987	2,069	48.2
Western Africa				
	Benin (2006)	17,794	13,486	5.9
	Ghana (2008)	4,916	2,95	16.5
	Guinea (2005)	7,954	6,327	5.4
	Liberia (2007)	7,092	4,508	10.5
	Mali (2006)	14,583	12,324	7.5
	Niger (2006)	9,223	7,431	7.1
	Nigeria (2008)	33,385	23,954	8.6
	Senegal (2005)	14,602	10,221	9.5
	Sierra Leone (2008)	7,374	5,373	7.9

Women were asked if they were currently using a method of contraception and what method they were using. The outcome was coded 1 if they were using a modern method (pill, IUD, injections, condom, male or female sterilization, implant, or diaphragm/foam/jelly) and 0 if they were using a traditional method, folkloric method, or were not currently using a method. The data were analyzed using the STATA 11.1 software package (College Station, Texas).

A separate multi-level logistic model was fitted for the outcome of modern contraceptive use in each country. Although the focus of this analysis was on community level influences, the models controlled for individual and household level variables that previous research has shown to influence contraceptive adoption. Indices were created to capture exposure to reproductive health messaging in the media, justification of violence, HIV knowledge, reproductive health knowledge, and decision-making autonomy. Bi-variate analyses were conducted between the individual and household level variables and the outcome of current contraceptive use (results not shown). Those variables significant in the bi-variate analysis were included in the model. Multicollinearity was checked and none was found, or where it was found, a decision to include only one of the variables in the model was made.

The hierarchical structure of the DHS data violates the assumption of independence as women are clustered within PSUs; if ignored, the standard errors are underestimated. A multi-level modeling technique was employed to account for the hierarchical structure of the data and allow for the estimation of community level influences on modern contraceptive use [16], [17]. Since the DHS does not collect community level data, community level variables were created by averaging individual level data to the PSU which serves as a proxy for the respondent’s community in this analysis and consists of approximately 30 households. The analysis controlled for urban and rural residence to account for possible differences in how communities are defined in urban and rural areas and the likelihood that PSUs in rural areas could more closely approximate communities because of more clearly defined clustering of households in less densely populated areas. Derived community level variables have been used previously to understand a range of reproductive health outcomes, including contraceptive use [8], [19], [21], [22], [23], [31]. Many of these studies used DHS data and derived variables using a similar method. While there are limitations to using derived variables and PSUs as proxies for community, in the absence of routinely collected community level variables, studies have depended on these derived variables to explore community level influences on health outcomes.

While some studies have begun to address the impact of the community environment on contraceptive use, they have either focused on the influence of the health care environment or focus on a single domain of the community. This study sought to expand the range of community level variables examined. Community level variables were chosen based on the findings of previous studies examining factors associated with modern contraceptive use in the African region and conceptualized into four domains: community demographics and fertility norms, community economic prosperity, community gender norms and inequalities, and community health knowledge and media exposure (Table 2).

**Table 2:** Operational definitions for community level variables used in modeling determinants of modern contraceptive use in 21 study countries.

COMMUNITY LEVEL VARIABLES	DEFINITION
**Community demographics and fertility norms**	
Mean age at marriage in the community	Mean age at marriage for women ages 15–49 in the community
Mean age at first intercourse in the community	Mean age at first intercourse for women ages 15–49 in the community
Mean age at first birth in the community	Mean age at first birth for women ages 15–49 in the community
Mean ideal number of children in the community	Mean ideal number of children in the community
Gender composition of children in the community	Ratio of boys to girls in the community, calculated as the number of living boys divided by the number of living girls.
**Community economic prosperity**	
Mean community wealth index factor score	Mean wealth index factor score, reflects ownership of durable goods and housing characteristics
**Community gender norms & inequalities**	
Mean community violence justification index score	5 point scale of attitudes towards domestic violence, lower score indicates that violence is not justified. Variables included in this index were the following: going out without telling the husband, neglecting children, arguing with the husband, refusing sex with the husband, and burning food. Some variation across countries with regard to questions included.
Mean community decision-making autonomy score	5 point scale of decision making autonomy where a higher score indicates higher decision making control. Variables included in this index were the following: final say on own health care, final say on making large household purchases, final say on making household purchases for daily needs, and final say on visits to family or relatives. Some variation across countries with regard to questions included.
Women in the community with at least a primary education	Proportion of women in the community with at least a primary education
Men in the community with at least a primary education	Proportion of men in the community with at least a primary education
Ratio of men to women employed in the community	Ratio of men employed in the community to women employed in the community (coded: 0 = no; 1 = yes)
**Community health knowledge and media exposure**	
Mean community HIV knowledge index score	7 point scale of knowledge of HIV where higher scores indicate greater knowledge of HIV. Variables included were the following: whether the respondent had heard of HIV/AIDS, three questions about reducing risk for infection (through abstinence, using condoms, and having just one uninfected partner who has not had other partners) and two questions regarding transmission (can people get AIDS virus from mosquitoes, can people get AIDS virus by sharing food with a person who has AIDS.
Mean community reproductive knowledge index score	5 point scale of reproductive health knowledge where higher scores indicate greater knowledge of reproductive health. Variables included were knowledge of the ovulatory cycle, knowledge of a contraceptive method, heard of AIDS or other STDs, heard of other STDs. Some variation across countries with regard to questions included.
Mean community media exposure index score	4 point scale of exposure to reproductive health messages in the media in the past month (radio, TV, and newspaper)

### Community Demographics and Fertility Norms (Table 2)

Attitudes towards fertility and childbearing prevalent in the community may shape individual contraceptive use by creating a normative expectation around the number of children in each family. Thus, women living in a community in which there is a general desire for a large number of children may feel social pressure to not use a contraceptive method. In addition, prevailing patterns of marriage, childbearing and sexual intercourse may represent social scripts that women are expected to follow and may represent the social and economic opportunities available to women [8], [33], [34], [35], [36]. While sex preference has not be studied as extensively in the African context as it has in other parts of the world, evidence suggests that there is a strong preference for male children in some contexts [36]. To measure community demographics and fertility norms, five variables were chosen: the mean age at marriage for women in the community, the mean age at first intercourse for women in the community, the mean age at first birth for women in the community, the mean ideal of number of children each woman would have in the community, and the gender composition of the children in the community. The gender composition of the children in the community was a ratio measure of the number of living boys in the community divided by the number of living girls. Values greater than 1 indicated more boys and values less than 1 indicated more girls.

### Community Economic Prosperity (Table 2)

Previous studies have explored the impact of increased household and community wealth on reproductive health indicators [9], [23], [37]. In particular, evidence suggests that wealth is associated with increased contraceptive use, possibly because of the greater potential to allocate scarce resources for reproductive health [9], [23], [37]. To measure community level wealth, the mean household index factor score was taken for each PSU. The wealth index factor score reflected ownership of durable goods and housing characteristics and has been shown to be an effective proxy for household wealth [38], [39].

### Community Gender Norms and Inequalities (Table 2)

Gender norms and inequalities in the community impact the level of decision-making autonomy experienced by women [6]. Educational attainment is often associated with increased access to social networks [7], [40], [41]. To measure community gender norms and inequalities, five variables were chosen: the mean community violence justification index score, the mean community decision-making autonomy score, the proportion of women in the community with at least a primary education, the proportion of men in the community with at least a primary education, and the ratio of men to women employed in the community. The ratio of men to women currently employed in the community was calculated by dividing the total number of men employed in the community by the total number of women employed where 0 = unemployed and 1 = employed. Information regarding employment status was taken from the Women’s Questionnaire where women reported their own current employment and that of their husband. The violence justification index was a 5 point scale of attitudes towards domestic violence where a lower score indicated fewer instances where the respondent justified violence. Decision-making autonomy was also measured as a 5 point scale where a higher score indicated more instances where the respondent made decisions alone.

### Community Health Knowledge and Media Exposure (Table 2)

Previous studies have demonstrated that increased health knowledge and exposure to health messaging in the media have a positive impact on reproductive health outcomes [8], [41]. Three variables were chosen to measure health knowledge and media exposure at the community level: mean community HIV knowledge index score, mean community reproductive health knowledge index score, and mean community media exposure index score. The index for HIV knowledge was a 7 point scale where a higher score indicated greater correct knowledge of HIV. The index for reproductive health knowledge was a 5 point scale where a higher score indicated greater knowledge of reproductive health. Finally, the media exposure index was a 4 point scale where a higher score indicated exposure to a greater number of sources of reproductive health messages in the media (radio, TV and newspaper).

An iterative model building process was used and a random intercept fitted to account for the hierarchical structure of the data. As women are nested within communities, they violate the basic assumption of independence. Furthermore, women in the same community are more likely to be similar than individuals in different communities. Fitting a random intercept allows for the estimation of inter-and intra-cluster variance. Model 1 only included individual and household level variables and model 2 included the addition of the community level variables. A likelihood ratio test was used to examine the significance of the addition of the community level variables. The likelihood ratio test served as a chunk test for the community level variables so that the individual and household level model (Model 1) could be compared to the full model (Model 2). The full model included the community level variables while controlling for the individual and household level variables in model 1. The difference between the two models, expressed as a p-value, can be obtained by taking the difference between the log likelihoods of each model (−2(log likelihood Model 1)– −2(log likelihood Model 2)). The difference of the log likelihoods is equivalent to a Chi Square test statistic, with degrees of freedom equal to the difference in the number of parameters between the 2 models. The p-value for the χ^2^ test statistic was obtained using the CHIDIST function in Excel which returns the one-tailed probability of the chi-squared distribution. The result was then doubled to obtain the two-tailed probability, a more robust test of difference. In addition to the likelihood ratio test, the sigma mu values for model 1 and model 2 were reported to show the remaining unexplained random variance in the two models for each country.

## Results

Prevalence of modern contraceptive methods varies significantly across the 21 study countries, from a low of 5.4% in Guinea to a high of 58.0% in Zimbabwe (Table 1). The focus of this analysis was on the associations between community level variables and contraceptive use and for the purpose of this study, individual and household level variables act as controls. The individual and household level results were not surprising. At the individual level and household levels, wealth and education were positively associated with contraceptive use. Parity was negatively associated with contraceptive use as women with fewer living children have an increased likelihood of currently using a contraceptive method. Age was significantly associated with contraceptive use. Overall, the greatest proportion of women using a modern contraceptive method was between the ages of 20 and 34, with some variation between countries. In general, community level effects associated with contraceptive use differed, with most variables showing a mixed effect across the study countries included (for example mean age at marriage in the community, mean community violence justification index score, and ratio of men to women employed in the community). There were, however, two variables in the community demographics and fertility norms domain that were more consistently associated with contraceptive use. The mean ideal number of children in the community was negatively associated with contraceptive use in 11 countries and mean age at first birth in the community which negatively associated with contraceptive use in 6 countries.

### Community Demographics and Fertility Norms (Table 3)

**Table 3:** Community level results of multilevel logistic model for the outcome of modern contraceptive use, domains of community demographics and fertility norms and community economic prosperity.

	Region and Country	Community demographics and fertility norms					Community economic prosperity
		Mean age at marriagein the community	Mean age at first intercourse in the community	Mean age at first birth in the community	Mean ideal numberof children in the community	Gender compositionof children in the community	Mean community wealth index factor score
**Eastern Africa**							
	Ethiopia (2005)	1.04 (0.89, 1.21)	0.92 (0.77, 1.09)	0.90 (0.81, 1.00)	**0.84 (0.78, 0.91)**	0.99 (0.75, 1.31)	1.00 (1.00, 1.00)
	Kenya (2008–09)	0.93 (0.85, 1.02)	1.09 (1.00, 1.20)	1.01 (0.91, 1.11)	**0.74 (0.65, 0.83)**	1.14 (0.91, 1.43)	1.00 (1.00, 1.00)
	Madagascar (2008–09)	1.08 (0.98, 1.18)	0.98 (0.89, 1.09)	**0.89 (0.81, 0.98)**	**0.68 (0.62, 0.74)**	1.08 (0.85, 1.37)	1.00 (1.00, 1.00)
	Rwanda (2005)	1.11 (0.90, 1.35)	0.92 (0.74, 1.13)	0.93 (0.76, 1.15)	**0.70 (0.58, 0.86)**	0.93 (0.66, 1.30)	1.00 (1.00, 1.00)
	Uganda (2006)	1.11 (0.98, 1.25)	0.97 (0.83, 1.13)	0.98 (0.85, 1.13)	0.90 (0.78, 1.05)	**1.48 (1.11, 1.97)**	1.00 (1.00, 1.00)
	Zambia (2007)	1.01 (0.91, 1.11)	1.10 (0.97, 1.26)	**0.80 (0.69, 0.91)**	0.81 (0.70, 0.92)	1.00 (0.75, 1.35)	1.00 (1.00, 1.00)
	Zimbabwe (2005–06)	**0.84 (0.77, 0.92)**	1.10 (0.97, 1.24)	1.02 (0.90, 1.14)	0.81 (0.70, 0.93)	0.93 (0.74, 1.18)	1.00 (1.00, 1.00)
**Middle Africa**							
	Congo (2005)	1.09 (0.99, 1.20)	0.96 (0.79, 1.16)	0.99 (0.88, 1.11)	1.11 (0.93, 1.32)	0.77 (0.55, 1.07)	1.00 (1.00,.100)
	Democratic Republic of Congo (2007)	1.14 (0.95, 1.36)	0.83 (0.69, 1.00)	0.90 (0.74, 1.09)	**0.83 (0.72, 0.94)**	0.66 (0.38, 1.16)	1.00 (1.00, 1.00)
**Northern Africa**							
	Egypt (2008)	1.06 (0.99, 1.13)	**	**0.92 (0.87, 0.99)**	**0.55 (0.50, 0.61)**	1.02 (0.89, 1.17)	**1.00 (1.00, 1.00)**
**Southern Africa**							
	Namibia (2006–07)	1.02 (0.98, 1.05)	1.03 (0.97, 1.08)	0.96 (0.91, 1.01)	**0.92 (0.85, 1.00)**	0.94 (0.79, 1.11)	1.00 (1.00, 1.00)
	Swaziland (2006–07)	0.96 (0.90, 1.02)	1.03 (0.92, 1.17)	0.97 (0.88, 1.08)	**0.77 (0.65, 0.93)**	1.08 (0.85, 1.38)	1.00 (1.00, 1.00)
**Western Africa**							
	Benin (2006)	1.09 (0.95, 1.25)	1.00 (0.89, 1.13)	**0.85 (0.75, 0.96)**	0.92 (0.82, 1.04)	0.89 (0.66, 1.19)	1.00 (1.00, 1.00)
	Ghana (2008)	0.97 (0.87, 1.07)	0.99 (0.88, 1.13)	1.08 (0.97, 1.20)	0.94 (0.82, 1.09)	1.10 (0.83, 1.45)	1.00 (1.00, 1.00)
	Guinea (2005)	**1.27 (1.02, 1.59)**	0.96 (0.73, 1.25)	**0.83 (0.71, 0.98)**	0.87 (0.68, 1.11)	**2.14 (1.25, 3.69)**	1.00 (1.00, 1.00)
	Liberia (2007)	0.96 (0.87, 1.06)	1.11 (0.94, 1.32)	0.98 (0.88. 1.10)	0.95 (0.83, 1.09)	0.89 (0.59, 1.32)	1.00 (1.00, 1.00)
	Mali (2006)	1.09 (0.91, 1.23)	1.14 (0.97, 1.34)	**0.87 (0.78, 0.98)**	0.91 (0.81, 1.02)	1.10 (0.78, 1.57)	**1.00 (1.00, 1.00)**
	Niger (2006)	0.95 (0.68, 1.32)	1.25 (0.87, 1.79)	0.82 (0.67, 1.01)	0.94 (0.82, 1.07)	0.85 (0.53, 1.34)	1.00 (1.00, 1.00)
	Nigeria (2008)	**0.88 (0.80, 0.96)**	**1.09 (1.01, 1.17)**	0.97 (0.88, 1.06)	**0.71 (0.66, 0.76)**	1.01 (0.79, 1.27)	1.00 (1.00, 1.00)
	Senegal (2005)	**1.22 (1.02, 1.45)**	**0.75 (0.62, 0.91)**	1.00 (0.88, 1.14)	**0.72 (0.62, 0.84)**	1.04 (0.74, 1.46)	1.00 (1.00, 1.00)
	Sierra Leone (2008)	0.97 (0.87, 1.09)	**1.24 (1.04, 1.49)**	0.92 (0.82, 1.03)	**0.76 (0.61, 0.94)**	1.38 (0.88, 2.15)	1.00 (1.00, 1.00)

Values reported as adjusted odds ratio (95% CI)*†.

* Models controlled for the following individual and household level factors: age, age at marriage, partner age difference, number of living children, death of a child, gender composition of children, religion, residence, wealth, employment, education (respondent and partner), violence index, decision-making autonomy index, HIV knowledge index, reproductive health knowledge index, media exposure index.

† Bolded figures are significant at 0.05 level.

** Information on age at first intercourse not collected in the 2008 Egypt DHS – Women’s Questionnaire.

Mean age at marriage in the community was negatively associated with using a modern contraceptive method in two countries (Nigeria OR 0.88 (0.80, 0.96) and Zimbabwe OR 0.84 (0.77, 0.92)) and positively associated with contraceptive use in two other countries (Guinea OR 1.27 (1.02, 1.59) and Senegal OR 1.22 (1.02, 1.45)). Similarly, the association between age at first intercourse and contraceptive use was mixed. In Nigeria and Sierra Leone, women in communities where there was a higher mean age at first intercourse had a greater likelihood of using a contraceptive method (OR 1.09 (1.01, 1.17) and OR 1.24 (1.04, 1.49) respectively) whereas in Senegal, women were less likely to use a contraceptive method in communities with a higher mean at first intercourse (OR 0.75 (0.62, 0.91)). Community mean age at first birth was significantly associated with contraceptive use in 6 countries. In Benin, Egypt, Ethiopia, Guinea, Madagascar, Mali and Zambia, women in communities with a higher mean age at first birth were less likely to use a contraceptive method (the effect was largest in Zambia (OR 0.80 (0.69, 0.91)) and the weakest in Egypt (OR 0.92 (0.87, 0.99)). The mean ideal number of children in the community was significantly associated with contraceptive use in more than half of all countries included in this analysis (DRC, Egypt, Ethiopia, Kenya, Madagascar, Namibia, Nigeria, Rwanda, Senegal, Sierra Leone, and Swaziland). Women in communities with a higher mean ideal number of children were less likely to use a contraceptive method (the effect size was greatest in Egypt OR 0.55 (0.50, 0.61) and weakest in Nigeria OR 0.92 (0.85, 1.00)). The gender composition of children in the community was significantly associated with contraceptive use in two countries. In communities where there were more living boys than girls, women were more likely to use a contraceptive method (Guinea OR 2.14 (1.25, 3.69) and Uganda OR 1.48 (1.11, 1.97)).

### Community Economic Prosperity (Table 3)

Community level wealth was significantly associated with contraceptive use in two countries. In both Egypt and Mali, wealth was significantly associated with contraceptive use (p-value <0.05) however the effect size was negligible (OR 1.00, (1.00, 1.00) in both countries).

### Community Gender Norms and Inequalities (Table 4)

**Table 4:** Community level results of multilevel logistic model for the outcome of modern contraceptive use, community gender norms and inequalities domain.

	Region and Country	Community gender norms & inequalities				
		Mean communityviolence justificationindex score	Mean community decision-making autonomyscore	Women in thecommunity withat least aprimary education	Men in the community withat least a primary education	Ratio of men to women employed in the community
**Eastern Africa**						
	Ethiopia (2005)	0.91 (0.79, 1.06)	1.11 (0.89, 1.38)	1.95 (0.90, 4.23)	0.93 (0.45, 1.89)	1.01 (0.99, 1.03)
	Kenya (2008–09)	**0.80 (0.71, 0.91)**	1.09 (0.91, 1.30)	1.25 (0.45, 3.50)	0.86 (0.24, 3.04)	0.94 (0.89, 1.00)
	Madagascar (2008–09)	0.98 (0.84, 1.15)	**1.23 (1.05, 1.43)**	0.82 (0.42, 1.59)	1.05 (0.52, 2.13)	0.96 (0.69, 1.33)
	Rwanda (2005)	1.22 (0.99, 1.51)	1.21 (0.98, 1.49)	2.39 (0.98, 5.82)	1.08 (0.45, 2.56)	1.01 (0.94, 1.09)
	Uganda (2006)	0.91 (0.79, 1.05)	0.98 (0.78, 1.22)	2.05 (0.87, 4.80)	2.01 (0.56, 7.27)	1.05 (0.82, 1.33)
	Zambia (2007)	**0.70 (0.62, 0.78)**	1.05 (0.85, 1.30)	1.42 (0.53, 3.81)	0.43 (0.13, 1.43)	**1.05 (1.01, 1.10)**
	Zimbabwe (2005–06)	**1.16 (1.01, 1.33)**	0.97 (0.82, 1.14)	0.76 (0.21, 2.80)	**5.12 (1.05, 25.01)**	1.00 (0.97, 1.02)
**Middle Africa**						
	Congo (2005)	1.00 (0.79, 1.28)	**	4.04 (0.86, 19.01)	0.27 (0.03, 2.78)	1.06 (0.86, 1.31)
	Democratic Republicof Congo (2007)	1.03 (0.84, 1.27)	0.98 (0.71, 1.35)	0.78 (0.22, 2.83)	0.49 (0.07, 3.37)	0.96 (0.85, 1.08)
**Northern Africa**						
	Egypt (2008)	**0.88 (0.82, 0.95)**	0.95 (0.86, 1.06)	1.20 (0.82, 1.75)	**0.58 (0.38, 0.87)**	**1.01 (1.01, 1.02)**
**Southern Africa**						
	Namibia (2006–07)	0.98 (0.87, 1.10)	1.01 (0.90, 1.15)	**2.25 (1.15, 4.40)**	0.75 (0.43, 1.32)	1.04 (0.99, 1.08)
	Swaziland (2006–07)	1.00 (0.75, 1.33)	0.98 (0.79, 1.22)	0.79 (0.31, 2.02)	0.59 (0.25, 1.40)	1.01 (0.95, 1.06)
**Western Africa**						
	Benin (2006)	1.08 (0.96, 1.21)	1.07 (0.91, 1.24)	0.69 (0.31, 1.55)	1.07 (0.58, 1.98)	1.18 (0.80, 1.75)
	Ghana (2008)	0.99 (0.81, 1.20)	1.07 (0.86, 1.33)	0.98 (0.43, 2.22)	0.53 (0.22, 1.24)	0.88 (0.58, 1.35)
	Guinea (2005)	1.03 (0.80, 1.34)	1.01 (0.73, 1.40)	0.71 (0.14, 3.59)	0.88 (0.25, 3.08)	1.04 (0.62, 1.74)
	Liberia (2007)	1.14 (0.97, 1.34)	1.05 (0.82, 1.35)	0.83 (0.32, 2.16)	0.75 (0.27, 2.07)	**1.10 (1.04, 1.17)**
	Mali (2006)	0.98 (0.87, 1.10)	1.09 (0.91, 1.31)	1.54 (0.62, 3.82)	**2.14 (1.02, 4.45)**	1.02 (1.00, 1.05)
	Niger (2006)	**1.21 (1.04, 1.40)**	1.23 (0.93, 1.61)	**7.20 (1.60, 32.48)**	0.37 (0.12, 1.13)	0.98 (0.94, 1.02)
	Nigeria (2008)	0.92 (0.84, 1.02)	1.01 (0.84, 1.20)	1.61 (0.78, 3.32)	1.06 (0.49, 2.30)	**0.91 (0.84, 0.99)**
	Senegal (2005)	0.98 (0.86, 1.10)	1.04 (0.80, 1.34)	1.15 (0.46, 2.85)	2.06 (0.96, 4.44)	**0.95 (0.91, 0.99)**
	Sierra Leone (2008)	0.95 (0.81, 1.11)	1.09 (0.79, 1.49)	1.94 (0.60, 6.32)	1.70 (0.62, 4.67)	1.08 (0.87, 1.34)

Values reported as adjusted odds ratio (95% CI)*†.

* Models controlled for the following individual and household level factors: age, age at marriage, partner age difference, number of living children, death of a child, gender composition of children, religion, residence, wealth, employment, education (respondent and partner), violence index, decision-making autonomy index, HIV knowledge index, reproductive health knowledge index, media exposure index.

† Bolded figures are signpone.0040670.g001.tifificant at 0.05 level.

** Information on decision-making autonomy not collected in the 2005 Congo DHS – Women’s Questionnaire.

The association between violence justification at the community level and use of a contraceptive method was mixed. In Egypt, Kenya, and Zambia, women in communities where violence was justified in more circumstances on average were less likely to use a contraceptive method (Kenya OR 0.80 (0.71, 0.91), Egypt OR 0.88 (0.82, 0.95), and Zambia OR 0.70 (0.62, 0.78)). Conversely, greater justification of violence at the community level was also associated with greater odds of contraceptive use in two countries (Niger OR 1.21 (1.04, 1.40) and Zimbabwe OR 1.16 (1.01, 1.33)). Community level decision making autonomy was significantly associated with contraceptive use in only one country. Women in communities with a higher mean decision-making autonomy score were more likely to use a contraceptive method in Madagascar (OR 1.23 (1.05, 1.43). Both men’s and women’s education were significantly associated with contraceptive use. Women in communities with a greater proportion of women who had at least a primary education were more likely to use a contraceptive method in two countries (Namibia OR 2.25 (1.15, 4.40) and Niger OR 7.20 (1.60, 32.48)). The association between men’s education and contraceptive use was mixed. In two countries, women in communities where a greater proportion of men had at least a primary education were more likely to use a contraceptive method (Mali OR 2.14 (1.02, 4.45) and Zimbabwe OR 5.12 (1.05, 25.01)). However, in Egypt, women in communities where a greater proportion of men had at least a primary education, women had a decreased likelihood of using a modern contraceptive method (0.58 (0.38, 0.87)). The association between employment and contraceptive use was conflicting. In three countries, women in communities with a greater ratio of men than women employed were more likely to use a contraceptive method (Egypt OR 1.01 (1.01, 1.02), Liberia OR 1.10 (1.04, 1.17), and Zambia OR 1.05, (1.01, 1.10)). However, in two countries, women in communities with a greater ratio of men than women employed were less likely to use a contraceptive method (Nigeria OR 0.91 (0.84, 0.99) and Senegal OR 0.95 (0.91, 0.99)).

### Community Health Knowledge and Media Exposure (Table 5)

**Table 5:** Community level results of multilevel logistic model for the outcome of modern contraceptive use, community gender health knowledge and media exposure domain.

	Region and Country	Community health knowledge and media exposure		
		Mean communityHIV knowledgeindexscore	Mean community reproductive knowledge index score	Mean community media exposure index score
**Eastern Africa**				
	Ethiopia (2005)	**1.40 (1.15, 1.70)**	0.92 (0.65, 1.30)	0.85 (0.60, 1.20)
	Kenya (2008–09)	1.10 (0.85, 1.42)	1.04 (0.70, 1.55)	0.85 (0.69, 1.04)
	Madagascar (2008–09)	**	††	**1.41 (1.07, 1.87)**
	Rwanda (2005)	0.82 (0.61, 1.11)	1.79 (0.98, 3.29)	0.68 (0.44, 1.05)
	Uganda (2006)	1.05 (0.83, 1.33)	1.29 (0.77, 2.19)	1.13 (0.74, 1.72)
	Zambia (2007)	1.12 (0.83, 1.50)	**0.39 (0.24, 0.64)**	1.35 (0.97, 1.89)
	Zimbabwe (2005–06)	1.03 (0.85, 1.26)	**1.55 (1.03, 2.34)**	1.07 (0.84, 1.37)
**Middle Africa**				
	Congo (2005)	1.25 (0.95, 1.64)	0.85 (0.55, 1.31)	1.46 (0.98, 2.18)
	Democratic Republic of Congo (2007)	1.18 (0.86, 1.63)	1.12 (0.70, 1.79)	**2.11 (1.08, 4.10)**
**Northern Africa**				
	Egypt (2008)	**	0.92 (0.81, 1.04)	1.12 (0.98, 1.27)
**Southern Africa**				
	Namibia (2006–07)	0.89 (0.76, 1.05)	1.21 (0.84, 1.73)	0.91 (0.74, 1.12)
	Swaziland (2006–07)	1.16 (0.86, 1.57)	1.99 (1.00, 3.96)	1.04 (0.78, 1.39)
**Western Africa**				
	Benin (2006)	0.91 (0.75, 1.10)	1.15 (0.84, 1.57)	1.10 (0.82, 1.48)
	Ghana (2008)	1.11 (0.86, 1.43)	1.03 (0.71, 1.50)	0.90 (0.63, 1.30)
	Guinea (2005)	0.86 (0.60, 1.24)	**1.89 (1.04, 3.45)**	**0.39 (0.22, 0.66)**
	Liberia (2007)	0.95 (0.79, 1.14)	1.40 (0.93, 2.10)	1.20 (0.78, 1.84)
	Mali (2006)	0.93 (0.78, 1.09)	1.33 (0.98, 1.79)	1.01 (0.77, 1.32)
	*Niger (2006)*	*0.79 (0.60, 1.04)*	***2.12 (1.35, 3.32)***	*0.65 (0.38, 1.10)*
	Nigeria (2008)	0.88 (0.78, 1.00)	0.83 (0.68, 1.02)	0.93 (0.77, 1.13)
	Senegal (2005)	0.91 (0.73, 1.13)	1.14 (0.75, 1.74)	0.86 (0.63, 1.16)
	Sierra Leone (2008)	0.86 (0.67, 1.10)	1.13 (0.81, 1.59)	1.52 (0.84, 2.76)

Values reported as adjusted odds ratio (95% CI)*†.

* *Models controlled for the following individual and household level factors: age, age at marriage, partner age difference, number of living children, death of a child, gender composition of children, religion, residence, wealth, employment, education (respondent and partner), violence index, decision-making autonomy index, HIV knowledge index, reproductive health knowledge index, media exposure index.*

† *Bolded figures are significant at 0.05 level.*

** *Information about HIV not collected in the 2008 Egypt DHS – Women’s Questionnaire; information about HIV only collected in sub-sample of the Madagascar 2008–09 DHS – Women’s Questionnaire, therefore excluded from this analysis.*

†† *Information about reproductive health only collected in sub-sample of the Madagascar 2008–09 DHS – Women’s Questionnaire.*

Community level knowledge of HIV was only significantly in one country. Women in communities with a higher mean HIV knowledge index score had greater odds of using a contraceptive method in Ethiopia (OR 1.40 (1.15, 1.70)). The association between community level reproductive health knowledge and contraceptive use was mixed. Women in communities with a higher reproductive health knowledge index score were more likely to use a contraceptive method in three countries (Guinea OR 1.89 (1.04, 3.45), Niger OR 2.12 (1.35, 3.32), and Zimbabwe OR 1.55 (1.03, 2.34)). In one country, however, women in communities with a higher reproductive health knowledge index score were less likely to use a contraceptive method (Zambia OR 0.39 (0.24, 0.64)). The mean community level media exposure to reproductive health messages was positively associated with contraceptive use in two countries and negatively associated with contraceptive use in one country. In the DRC and Madagascar, women in communities with a higher mean community media exposure index score had greater odds of using a modern contraceptive method (OR 2.11 (1.08, 4.10) and OR 1.41 (0.107, 1.87) respectively). Women in communities with a higher mean media exposure index score had a decreased likelihood of using a modern method of family planning in Guinea (OR 0.39 (0.22, 0.66)).

At the 0.05 alpha level, the results of the likelihood ratio test showed that in all but 5 of the 21 study countries, the addition of the community level variables as a chunk were significantly associated with the outcome of contraceptive use (Table 6). In all countries, there was a decrease in the sigma mu from the individual and household level model to the full model including the community level variables. A significant decrease in the sigma mu would suggest that the addition of community level variables accounts for some (or a significant proportion) of the unexplained random variance.

**Table 6:** Comparison of individual and household level model to community level model, sigma mu and likelihood ratio test p-value reported for 21 study countries.

	Region and Country	Individual Level Model Sigma Mu (SE)^1^	Community Level Model Sigma Mu (SE)^1^	Likelihood Ratio Test(p-value)^2^
**Eastern Africa**				
	Ethiopia (2005)	0.73 (0.06)	0.66 (0.06)	<0.0001
	Kenya (2008–09)	0.50 (0.06)	0.39 (0.06)	<0.0001
	Madagascar (2008–09)	0.70 (0.04)	0.60 (0.04)	<0.0001
	Rwanda (2005)	0.63 (0.09)	0.54 (0.09)	0.0003
	Uganda (2006)	0.46 (0.07)	0.40 (0.07)	<0.0001
	Zambia (2007)	0.64 (0.06)	0.47 (0.06)	<0.0001
	Zimbabwe (2005–06)	0.47 (0.06)	0.38 (0.06)	0.0001
**Middle Africa**				
	Congo (2005)	**0.17 (0.16)**	**0.003 (0.028)**	0.4253
	Democratic Republic of Congo (2007)	0.72 (0.08)	0.64 (0.08)	0.0081
**Northern Africa**				
	Egypt (2008)	0.59 (0.03)	0.45 (0.03)	<0.0001
**Southern Africa**				
	Namibia (2006–07)	**0.13 (0.18)**	**0.003 (0.022)**	0.0001
	Swaziland (2006–07)	**0.003 (0.030)**	**0.002 (0.019)**	0.2863
**Western Africa**				
	Benin (2006)	0.56 (0.07)	0.54 (0.07)	0.6813
	Ghana (2008)	0.54 (0.10)	0.52 (0.11)	1.0000
	Guinea (2005)	0.76 (0.10)	0.64 (0.10)	0.0062
	Liberia (2007)	0.48 (0.10)	0.42 (0.11)	0.3057
	Mali (2006)	0.42 (0.06)	0.36 (0.07)	0.0002
	Niger (2006)	0.69 (0.09)	0.58 (0.09)	0.0001
	Nigeria (2008)	0.72 (0.04)	0.58 (0.04)	<0.0001
	Senegal (2005)	0.61 (0.06)	0.47 (0.06)	<0.0001
	Sierra Leone (2008)	0.71 (0.11)	0.58 (0.11)	0.0027

^1^ *Models where variables account for all the PSU level variation are bolded.*

^2^ *Two-tailed p-value was used.*

## Discussion

The results of this analysis demonstrate that there is no single community influence on contraceptive use. Rather, communities influence contraceptive use through prevailing fertility norms, gender inequalities, health knowledge, and exposure to family planning messages in the media. The community level factors associated with contraceptive use vary across the 21 countries included in this analysis. This variation highlights the uniqueness of country specific contexts and demonstrates the range of community level factors that shape contraceptive uptake in the African region. Measures of community level demographics and fertility norms surfaced as most commonly associated with contraceptive use across the study countries. The mean ideal number of children in the community and the mean age at first birth for women in the community were negatively associated with contraceptive use in those countries where there was a significant effect (11 countries for the mean ideal number of children in the community and 6 countries for the mean age at first birth). These results emphasize that women seem to be influenced in their contraceptive choices by the fertility norms of their community environment and expectations around family size.

Within the domain of community gender norms and inequalities, the results showed greater variation in the impact of community level factors across countries. Attitudes towards violence may impact women’s autonomy and ability to seek health services and may reflect greater gender inequalities [29]. Furthermore, in places where women use contraceptives clandestinely, women may fear violence if they are discovered [25]. The impact of men’s education at the community level was mixed in the 3 countries where it was significantly associated with women’s contraceptive use, suggesting that increases in men’s educational attainment do not necessarily coincide with greater opportunities for women and may even result in greater gender inequalities. Increases in women’s educational attainment, were positively associated with a greater likelihood of using a contraceptive method in the two countries where the association was statistically significant. This may point to the role that education plays in expanding women’s networks and allowing them to build greater social capital in these settings. Similarly, living in a community where there was a more equal ratio of men to women employed could be positively associated with contraceptive use because women’s decision-making power and ability to allocate family resources for individual health needs may increase as their economic dependence on other family members decreases. Taken together, the effects of violence, men’s educational attainment, and employment were mixed, once again underscoring the differences between country contexts but highlighting the importance of gender equity at the community level in shaping contraceptive use uptake.

In general, the results of this analysis confirm the findings of previous community level studies with regard to the impact of health knowledge and exposure to family planning messages in the community [6], [8], [42]. It is probably not knowledge itself that impacts contraceptive use as evidence suggests that knowledge alone does not translate into use [43]. Instead, health knowledge may serve as a surrogate for the presence of health programs and greater exposure to health care services in the community. Increased exposure to family planning messages in the media could normalize contraceptive use at the community level and may create a more enabling environment for uptake of contraceptives in the 2 countries where exposure to media messages was positively associated with contraceptive use [6].

The community level variables included in this analysis were significantly associated with contraceptive use in all but 5 of the study countries (as demonstrated by the results of the likelihood ratio test) and accounted for a portion of the unexplained variance remaining in the individual model (as seen in the decrease in the sigma mu from model 1 to model 2). However, they do not fully account for the community level variation in contraceptive use. A limitation of this research is the inability to control for the presence of health care services in the community. It is possible that by controlling for health infrastructure level variables as well, more of the remaining variance could have been explained. Another limitation is the conceptualization of community. For this analysis, the PSU was used as proxy for the respondent’s community and community level variables were derived from individual level responses in the PSU. This is a geographic representation of community which may or may not correspond to the social dynamic of the community in its entirety. However, given the paucity of standardized data collected at the community level, using the PSU as a measure of community is the best approximation available. The results, therefore, also highlight the need to collect data at the community level instead of relying on derived community level variables and geographic definitions of community. The breadth and variation in community level variables found to be significantly associated with contraceptive use in this analysis, demonstrate the need to routinely collect community level data. This study can then serve as a starting point for future studies through suggesting pathways of community level influence that should be investigated further. With regard to the conclusions that can be made from this study, it is important to note that since the data used in this analysis were cross-sectional, temporality of the associations found cannot be determined.

### Conclusion

This study is the first of its kind as it includes a broader range of community factors and focuses on the entire African region. The results contribute to a deeper understanding of the community level factors that shape contraceptive use and confirm the importance of investigating community level influences and the need to focus on the role of place as it shapes reproductive health outcomes. Interventions aimed at impacting community norms regarding contraceptive use could be strengthened through integrating community dialogues and providing opportunities for women to more openly discuss issues related to reproductive health. When examining health behaviors, a stronger focus needs to be placed on factors beyond the individual and household levels and data collection should include community level variables. The findings of this innovative study highlight a range of community level factors that should be considered when planning public health interventions for increasing access to and utilization of modern contraceptive methods. In addition to strengthening the health-systems level response to unmet need for contraceptives, programs need to be sensitive to prevailing fertility and gender norms operating at the community level and tailor family planning messaging and programmatic efforts to maximize impact on women’s empowerment.
